# Increasing Melanoma—Too Many Skin Cell Damages or Too Few Repairs?

**DOI:** 10.3390/cancers5010184

**Published:** 2013-02-18

**Authors:** Örjan Hallberg, Olle Johansson

**Affiliations:** 1 Hallberg Independent Research, Brattforsgatan 3, 123 50 Farsta, Sweden; 2 The Experimental Dermatology Unit, Department of Neuroscience, Karolinska Institute, 171 77 Stockholm, Sweden; E-Mail: olle.johansson@ki.se

**Keywords:** melanoma, incidence, mortality, DNA damage, DNA repair, radiation, problem

## Abstract

Skin melanoma rates have been increasing for a long time in many Western countries. The object of this study was to apply modern problem-solving theory normally used to clear industrial problems to search for roots and causes of this medical question. Increasing cancer rates can be due to too many cell damage incidents or to too few repairs. So far, it has been assumed that the melanoma epidemic mainly is caused by increasing sun tanning habits. In order to explore this problem in more detail, we used cancer statistics from several countries over time and space. Detailed analysis of data obtained and a model study to evaluate the effects from increased damages or decreased repairs clearly indicate that the main reason behind the melanoma problem is a disturbed immune system. The possibility to introduce efficient corrective actions is apparent.

## 1. Introduction

In 1998 one of the authors, Örjan Hallberg, had been working for many years as quality manager and more recently as environmental manager within the Ericsson Corporation. The reliability performance of telecom products was carefully followed all over the World to watch out for early signs of increasing failure rates. Normally electronic products behave very well and after the first few years of use failures hardly ever happened.

But in the few cases when customer complaints started to increase for a specific product, this indicated a possibly very expensive problem, where e.g., 100,000 products from all over the World had to be called back for repair and/or replacement. Increasing failure rates were always treated as a warning sign of a very serious and expensive problem that immediately had to be acted upon in a professional way as quickly as possible.

**Figure 1:**
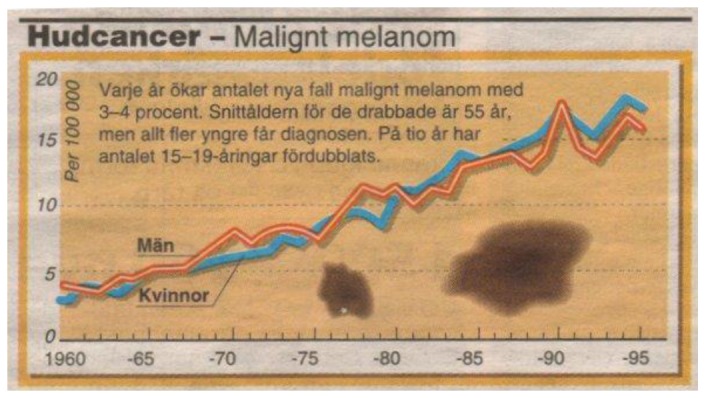
Increasing rates of melanoma in Sweden according to an article in Aftonbladet, 1998.

On 27 April 1998 a Swedish newspaper published a graph (see Figure 1) that caught the interest of Mr. Hallberg. The graph showed how a skin disease, melanoma, had been continuously increasing year by year since 1960 and that there seemed to be no levelling off in sight. Was this a sign of a health problem that should be dealt with in a professional way as any other industrial problem normally does?

The article did not mention any ongoing activity to address this obvious problem of national size. Instead it was mentioned that the increase probably was due to changing and increasing sun-tanning habits. It is recognized that the melanoma risk depends on intrinsic (genetic) and extrinsic (environmental) factors. Sun exposure is a relevant factor regarding creation of new cell damage, but less is known about factors that may reduce cell damage repair efficiency.

Hallberg got interested in this topic from a pure problem-solving point of view. Why not also use the standard procedure to address technical problems when it comes to public health issues? Actually, there would be lots of money to save in case the root cause was found and relevant corrective actions were taken by the responsible authorities. This was the first step on a long journey towards a better understanding of cancer epidemiology to start doing something, not only continue an endless research effort where every published paper ends with the statement “more research is needed in this field”.

## 2. Results—Problem Solving Theory Applied to Melanoma

There are good books written about problem solving. One example is *Rational Thinking* by Kepner and Traegö [1]. When a problem is reported there are a number of standard steps one should follow. The main steps in problem solving are:

1. State the problem
2. Specify facts about the problem
3. Identify possible causes
4. Evaluate possible causes
5. Confirm true cause

We will here follow those steps in detail and sum up with discussion and conclusions.

### 2.1. The Problem

The problem we will address is the fact that skin melanoma has become one of the fastest increasing cancers in Western countries, and we want to find out WHY?

### 2.2. Facts about the Melanoma Epidemic

- **What** are the characteristics of the problem?
  - Before
  - After
- **Where** has the problem been noticed?
  - What is specific with those areas?
  - Where has the problem **not** been noticed?
  - What are the differences between those areas?
- **When** was the problem at first noticed?
  - What happened then?
- **How** large is the problem?

In the case of the melanoma epidemic; a systematic analysis according to the standard problem solving procedures seems lacking in the medical scientific literature. Instead it is just assumed that changed sun-tanning habits were the true cause and that no deeper analysis of reasons behind this national catastrophe was necessary. Here we will address those questions one by one and give evidence on data by plots; sometimes copied from already published papers.

#### 2.2.1. What Are the Characteristics of the Problem?

Skin melanoma is a cancer that normally takes many years to develop. Initial skin damages, e.g., due to DNA damage caused by UV-radiation from the sun, may develop decades later into melanoma in the skin and then also spread to other parts of the body. Under normal conditions the body can cope with such damages quite well, and damages older than, say, 30 years should have been repaired or disposed by the immune system. The melanoma problem is characterized by a suddenly increasing incidence from about 1955 in the Nordic countries. If increasing rates were caused by increasing skin damages from UV radiation, this exposure must have started almost stepwise some decades earlier affecting all ages.

##### 2.2.1.1. What Were the Characteristics of Melanoma before the Problem Started?

**Figure 2:**
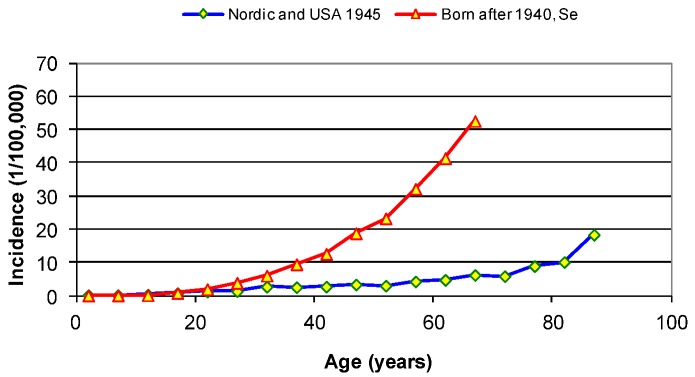
Melanoma incidence versus age in the Nordic countries and USA before 1950. Also shown is the melanoma incidence of birth cohorts born in 1940 and later in Sweden. From [2].

Before 1955 the rate of melanoma stayed under 5/100,000 person years (py) for all ages below 80 years, where it might increase up to 10. Figure 2 shows the age-specific rates in several countries before and after 1955. For both plots the incidences at ages below 15 years stay close to zero.

##### 2.2.1.2. What Were the Characteristics of Melanoma after the Problem Was Noticed?

**Figure 3:**
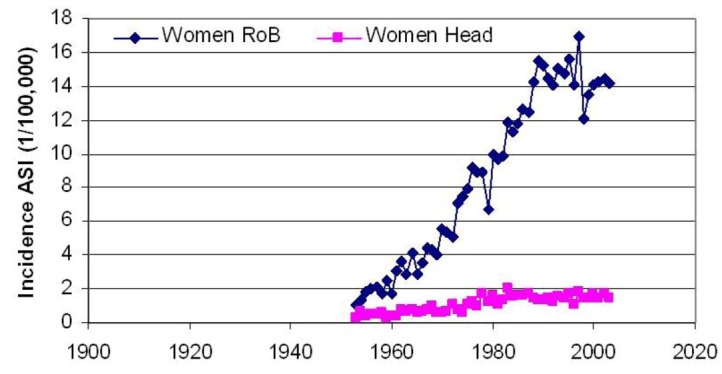
The incidence of melanoma in women in Norway roughly doubled on the head region after 1955 while it increased by almost 20 times on the rest of the body (RoB) [3].

At the beginning of the 20th century, melanoma was mainly found on sun-exposed areas of the body such as on the head or sometimes even on the feet. But after 1955 melanoma increased fastest on normally non-exposed parts of the body, see Figure 3. Melanoma on the head and on the rest of the body are often different in nature, as head melanomas are invariably associated with solar elastosis and chronic sun damage to the skin. The data in Figure 3 refer to the term melanoma in general, as used in databases.

Furthermore, as shown in Figure 2, the incidence did not level off after the age of 30 as it did earlier, but continued to increase by age for all persons born after 1940. 

#### 2.2.2. Where Has the Problem Been Noticed?

**Figure 4:**
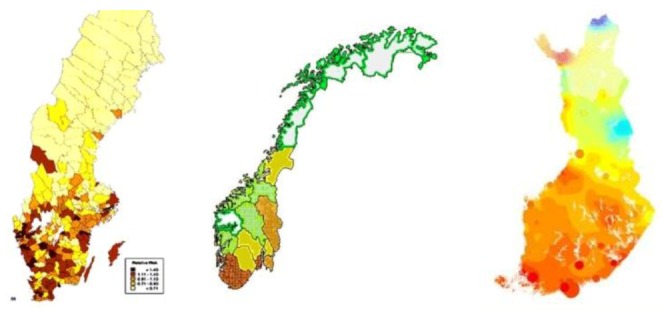
The incidence of melanoma is unevenly distributed within Nordic countries. The maps represent Sweden, Norway and Finland from left to right.

In Sweden the incidence of melanoma has been higher at lower latitudes and less in the northern parts of the country. The highest incidence has been noticed in a municipality in the middle of the country, Ödeshög. Figure 4 shows a map of melanoma incidence in Sweden as reported in 1996. Similar types of melanoma maps are found also in e.g., Norway and Finland.

##### 2.2.2.1. What Is Specific with Those Areas?

**Figure 5:**
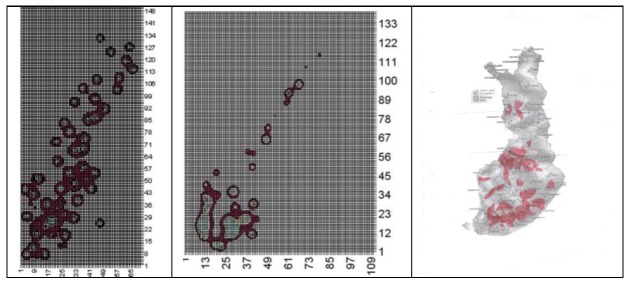
Calculated radiation levels from main broadcasting transmitters in Sweden, Norway and Finland. The first graph represents Sweden and shows areas with highest radiation levels. The second plot represents Norway, showing the highest levels in the south part of the country. The third plot gives areas in Finland covered by three or more main broadcasting transmitters (red colour).

In all three countries melanoma seems higher in coastal areas and especially in the southern parts. These areas are also more densely populated and urban compared to the more rural areas up north showing low melanoma incidence. Urban areas also include more cars and in general more environmental pollution. These areas are also more exposed to electromagnetic radiation from different sources than rural areas are. Figure 5 shows a plot of the radiation from main broadcasting transmitters in Sweden that to some extent looks similar to the melanoma map in Figure 4. The same applies also to Norway and Finland.

##### 2.2.2.2. Where on the Body Is Melanoma most Frequently Found?

**Figure 6:**
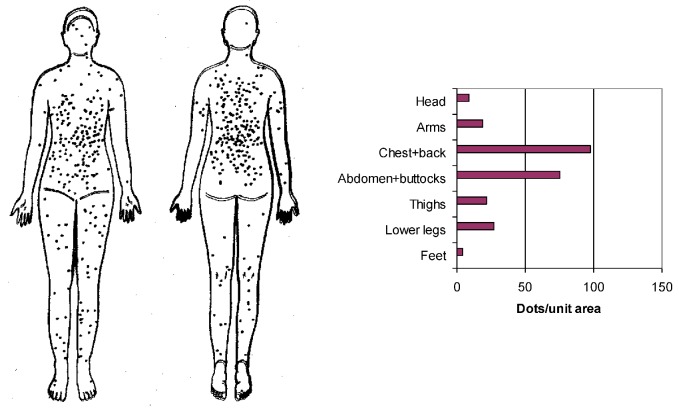
Distribution of melanoma over the body and the relative rate of melanoma per unit body area. Data are from Sweden and the details are from two doctoral thesis publications further referred to in [4].

Figure 3 shows that melanoma has been increasing mainly on the rest of the body, apart from the head region. Figure 6 gives more specific information on where melanoma is found; on the central parts of the body and specifically on the widest parts of the body [4].

#### 2.2.3. When Was the Problem at First Noticed?

**Figure 7:**
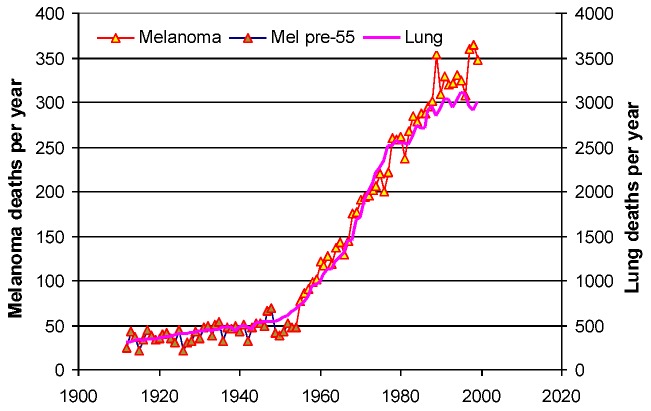
The number of deaths in skin melanoma and in lung cancer started to increase quite abruptly from 1955 in Sweden [5]. Data earlier than 1952 were obtained from paper files at Statistics Sweden (SCB).

According to Figure 1 it looks as the incidence may have been around 2–3/100,000 before 1960. As the Swedish cancer registry did not start until 1958 we had to go to paper files at Statistics Sweden to get earlier data. Figure 7 shows e.g., that the number of deaths due to both melanoma and lung cancer suddenly started to increase right after 1955, and it is striking how these two diseases generate deaths in quite a parallel way.

According to Figure 7 it is also noticeable that for every melanoma death there are 10 lung cancer deaths in Sweden. We looked at several other countries and found the same trend; melanoma and lung cancer seem to follow each other. In Norway there are eight lung cancer deaths per melanoma case, while in Denmark the ratio is 13 to one. Similar graphs can easily be plotted by use of cancer mortality registries like the WHO data base [6].

In the Nordic countries Sweden, Norway, Finland and Denmark it seems that melanoma started to increase from around 1955, while in Iceland it took off a number of years later. Thus the problem started in 1955 for the four Nordic countries. In the USA the melanoma rate had been increasing slowly since the 50s but the real and fast increase did not start until around 1973.

##### 2.2.3.1. What Happened Then?

The post-war era started lots of developments that tended to revolutionise our way of living. Nylon socks, plastic materials, electronic products added new flavour to the society. Right after the war the sales of private cars accelerated in a way that caused lots of traffic accidents and killed people, since virtually all drivers were inexperienced and had to learn from their mistakes.

Since the pale period of the 30s when sun tanning was more a sign of the working class, a new way of appreciating the healthy sunshine was developing. At that time sun-tanning was not associated with skin cancer at all and it has been estimated that sun-tanning was increasingly used by the population from 1930 up to 1980, when the sun-cream industry and authorities started to warn about the dangers of UV-radiation emitted by the Sun.

More specifically to the year 1955 was the fact that a quite new system for broadcasting of FM-radio and TV channels started to roll out. It took about 10 more years before the whole country was covered by these around 60 main transmitter stations in Sweden. As a matter of fact, the same was the case for Norway, Finland and Denmark. In Sweden, Finland and Denmark the authorities set a maximum output power of 60 kW for FM transmitters, while Norway accepted up to 250 kW, just as is accepted in the USA.

**Figure 8:**
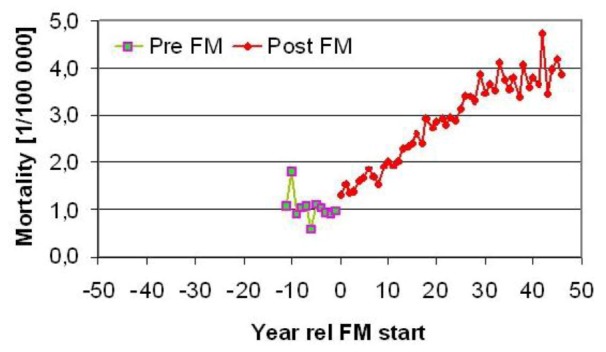
Melanoma mortality in Swedish counties related to the year they got FM radio broadcasting (from 1955 to 1965). From [7], with permission to copy.

As mentioned before, some counties of Sweden had to wait up to 10 years before the FM broadcasting system had been installed. It is thus interesting to see if the melanoma incidence stayed low and stable before they got the new radio or not. Figure 8 shows that this certainly was the case and is a strong argument for deeper studies.

#### 2.2.4. How Large Is the Problem?

**Figure 9:**
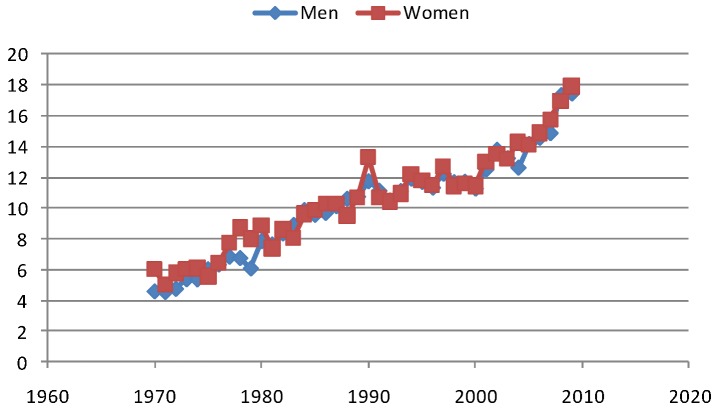
Age-standardized rate of melanoma in Sweden (world standard).

**Figure 10:**
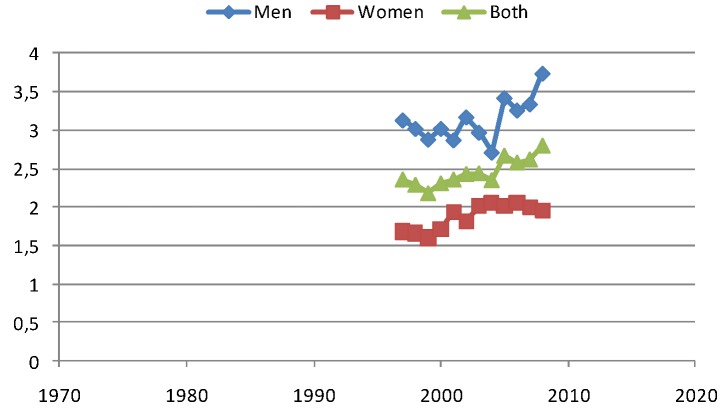
The mortality in melanoma starts to increase again after 2005 for men in Sweden.

Melanoma is a disease that mainly is a problem in Western types of developed countries. In Japan e.g., the melanoma incidence is only around 3% of the Swedish level, although Japan is a modern and well developed country. In Sweden the world age-standardized rate of melanoma is today approaching 20/100,000 py (see Figure 9). The incidence seems to have been levelling off around the year 2000, but has been increasing fast since 2005. This increase during the last years cannot be due to better and more observant doctors, since the mortality also starts to increase remarkably after 2005, especially for men (see Figure 10).

### 2.3. Possible Causes

Figure 7 gives a strong indication that we should look for a cancer stimulation factor that affects both melanoma and lung cancer, and if there is such a factor, other cancer forms might also be affected. Three of the above mentioned factors; sun-tanning habits, private car use and the roll-out of public radio and TV broadcasting need to be further evaluated to see to what degree they can explain the drastic increases seen in cancer incidence and mortality.

### 2.4. Evaluate Possible Causes

The increasing rate of melanoma has most often been devoted to changing sun-tanning habits. Here we will evaluate to what degree three different hypotheses can explain the noticed facts about the increasing rate of melanoma.

#### 2.4.1. Increasing Use of Private Cars

In this case we hypothesise that increasing use of cars is causing the melanoma explosion. We will then relate the annual number of cars added to the car pool with the number of new melanoma cases reported each year. It is possible to extract parameters of a characteristic function that best fits calculated to reported cases over time. The procedure to extract such a characteristic function out of growing populations was first described by Oscarsson and Hallberg [8].

**Figure 11:**
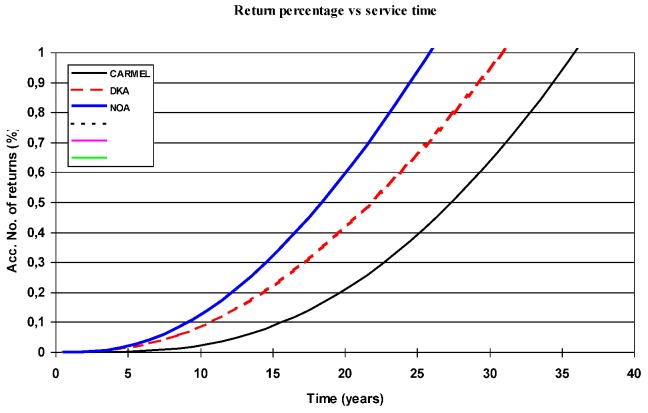
Optimum fit functions to correlate car population with melanoma incidence in Norway, Denmark and Sweden. The result indicates no similarity at all between cars and melanoma in the different countries.

Similar sets of data were collected from Sweden, Norway and Denmark to extract their respective characteristic functions. If the hypothesis is valid, we should expect to arrive at basically one and the same function for all countries. This analysis was performed in 2002, and the result was negative; the characteristic functions were different for all countries and therefore the association between melanoma and car population can be classified as a confounder, see Figure 11, where returns (%) refer not to faulty cars but instead to melanoma cases. According to the graph 10,000 new cars in Sweden one year should have caused 0.2% or 20 melanoma cases after 20 years, as an example.

#### 2.4.2. Immune Disturbance by Body-Resonant Broadcasting Radiation

In the mid-1950s the new broadcasting standard using frequency modulated (FM) carrier waves was introduced in Scandinavia. It took around 10 years before over 95% of the population was covered by this system, so some areas did not get this new system until about 1965. The frequency used for the FM radio is special, in the sense that the half wavelength fits well to the length of a human body. According to the WHO the bandwidth used for FM-radio makes the body to absorb up to ten times more energy than other bandwidths do. More specifically, this radiation is horizontally polarized, *i.e*., the electric field wants to drive currents in a horizontal direction. 

The hypothesis is now that, during sleep, the body might be positioned in a resonant direction and that weak induced currents all night, year after year may disturb the immune system in its normal, endless work to detect and repair or kill damaged skin cells. 

**Figure 12:**
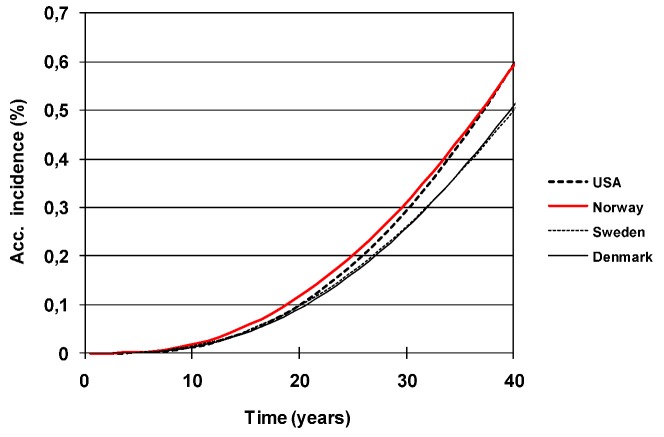
Characteristic functions for Exposure-Time-Specific-Incidence from FM-radiation in Sweden, Norway, Denmark and the USA [5].

A detailed analysis of the roll-out of the FM-radio system was performed for Sweden, Norway, and Denmark and for the USA. The number of people that became exposed for the new environment per year was used in combination with data on melanoma incidence, and the characteristic function was extracted for each country. The result is shown in Figure 12, and we can note that the function became basically identical for all countries despite the fact that there were four completely different sets of data.

Denmark and Sweden have exact the same response function, and Norway and the USA have also identical response, somewhat faster than Sweden and Denmark. It should be noticed that Sweden and Denmark have a maximum power limit of 60 kW from main FM-transmitters, while Norway and the USA accepts up to 250 kW.

**Figure 13:**
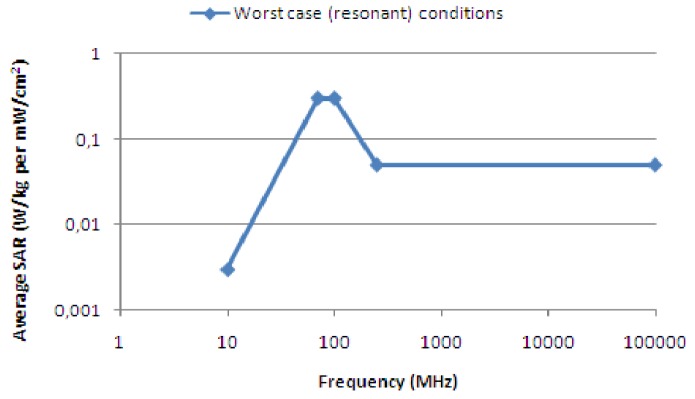
Radiation absorption by the human body *vs*. frequency [9].

Figure 13 shows that the FM band 87–108 MHz is classified by the WHO as the most powerful frequency band when it comes to radiation absorption [9]. In their fact sheet #304 WHO states: “In fact, due to their lower frequency, at similar RF exposure levels, the body absorbs up to five times more of the signal from FM radio and television than from base stations. This is because the frequencies used in FM radio (around 100 MHz) and in TV broadcasting (around 300 to 400 MHz) are lower than those employed in mobile telephony (900 MHz and 1800 MHz) and because a person's height makes the body an efficient receiving antenna. Further, radio and television broadcast stations have been in operation for the past 50 or more years without any adverse health consequence being established”.

**Figure 14:**
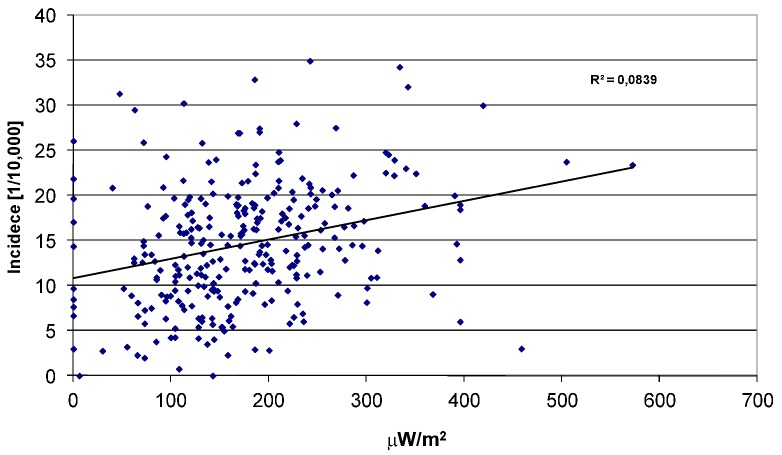
Calculated power density from main FM-transmitters *vs*. melanoma incidence in the 298 communities in Sweden.

If it mainly is the power density that determines the melanoma risk, we should then expect to find the highest melanoma rates in areas receiving the highest combined power density from surrounding main transmitters. Software was developed to calculate this power density over a country, see examples given in Figure 4 for Sweden and Norway. The calculated power densities were then correlated with reported melanoma rates in Figure 14. As can be seen, the correlation is very weak and the power density can be dismissed as a main factor for melanoma.

**Figure 15:**
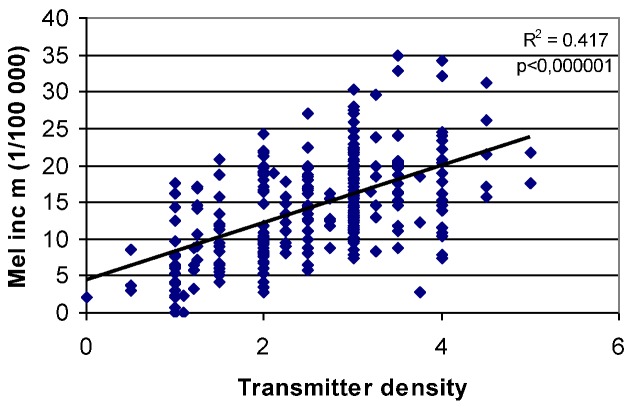
Melanoma incidence *vs*. the number of covering FM radio transmitters in Sweden [10].

If, instead, the most critical factor is body resonance, then the probability of sleeping in a resonant direction would very much depend on the number of surrounding transmitters. The half-wave length at the frequency 87 MHz is e.g., 1.74 m, which matches the human body length quite well. In case you are sleeping on a metal spring mattress which acts as a radio antenna, there is a risk that your body will constantly carry currents caused by reflected and standing waves during the whole night, year after year. Figure 15 gives the correlation between this number of surrounding main transmitters and the melanoma incidence in the same 289 communities of Sweden. Obviously, there is a very strong correlation, and the hypothesis cannot be dismissed. It is interesting to note, that the municipality having the highest melanoma incidence (35) in Figure 15 is also in the top three when the same analysis is done for breast cancer.

**Figure 16:**
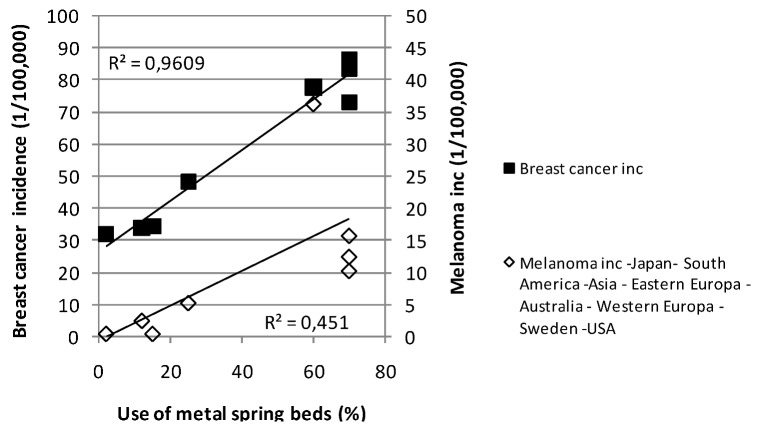
Melanoma and breast cancer incidence *vs*. the use of metal spring beds in different parts of the world, from [11].

The body-resonant hypothesis gets strong support from Figure 13 and Figure 15. If this is a major factor behind the melanoma increase we should examine what technical factors, beside bed direction, may be of importance to enhance the effects of body-resonant radiation. One obvious such factor is the bed structure. A metal spring mattress is acting as a TV antenna and will definitely increase the risk for standing waves and body currents that can disturb the immune system. Consequently, countries where such beds are less frequently used should be expected to show lower melanoma rates. Figure 16 reports on a review of bed standard and cancer in different areas of the world. Again, the data seems in favor of this hypothesis.

**Figure 17:**
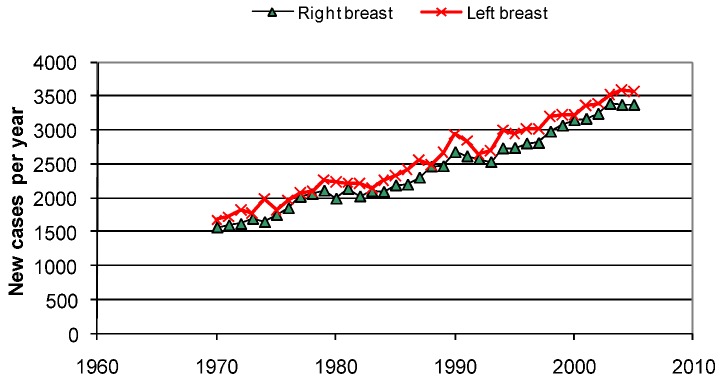
The number of new cases of breast cancer per year in Sweden shows a left dominance [13,14].

According to radio engineering expertise, standing waves reflected from metal structures may result in the highest field strength a quarter of a wavelength above the structure. Since people tend to sleep for longer time on their right side than on their left side we should therefore expect that breast cancer and melanoma should be slightly more prevalent on the left side of the body. This is, as a matter of fact, true and has been known for many years while an explanation has been lacking for decades. The left laterality in Sweden and many other western countries is around 10%, see Figure 17. In Japan, on the other hand, there is no left dominance of breast cancer reported; instead there is a slight right laterality of 5%, in line with their sleeping habits on non-metal Futons on the floor [11]. The incidence of melanoma in Japan is only around 3% of the rates noticed in Sweden. This difference can hardly be explained by skin color difference, since Japanese people who move to Western countries tend to get increased cancer rates within two generations.

“For virtually all cancers, with the passage of time, or in succeeding generations, rates tend to approach those of the native-born in the country of adoption”. “… and each has been hypothesized to have an environmentally determined hormonal or dietary component to its etiology” [12]. Since the mortality in breast cancer, melanoma and lung cancer is increasing exponentially by time since 1955 among elderly this cannot be explained by over-diagnosis.

#### 2.4.3. Skin Damage Due to Increasing Sun-Tanning Habits

**Figure 18:**
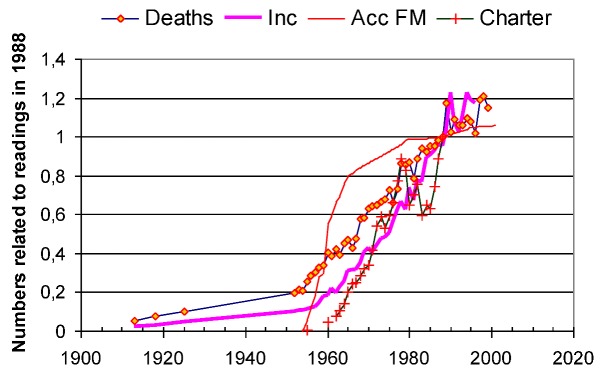
Charter travel, melanoma incidence and melanoma mortality start to increase from years 1962, 1958 and 1955 respectively [6].

Health authorities, sun cream industry and radiation safety authorities all state that it is the increasing exposure to UV radiation from the Sun that is the main cause of the melanoma epidemic. Looking into the increasing rates of charter travels, melanoma incidence and mortality, gives however a contradictory picture. Travel started in 1962, and then the melanoma incidence began to increase from 1958, followed by an increasing mortality from 1955. Figure 18 does not support the hypothesis of a sun-tanning-caused melanoma epidemic.

### 2.5. Confirm True Cause

Out of the three hypotheses presented in Section 2.4, only one, the hypothesis of body-resonant radiation and a disturbed immune system, seems to match collected facts. If that is a good hypothesis about the cause to this disease, it should be possible to model the process in mathematical terms so that future trends can be predicted.

#### 2.5.1. Designing a Melanoma Model

We assume that skin damages caused by e.g., UV radiation exposure from the sun have a certain probability to develop into melanoma over time. For practical reasons all damage collected during one year will be treated as the damage dose for that year, from which the melanoma risk will be modelled over time. The next year a new dose of damage will be added that contributes to the total body-risk of getting melanoma and so on.

But Mother Nature would not allow the sun to kill animals and humans, so this melanoma risk is coped with by a good and effective immune system that repairs damaged cells or, if they are too damaged, just kills them out of mercy. In summary, the model is compiled by a Life Matrix taking both the melanoma basic risk and the repair efficiency into account. Table 1 shows the Life Matrix.

The risk function F_i_ is the risk contribution during year **i** from the product of the basic cancer risk C_i_ and the fraction of unrepaired damages (1-R_i_). Both C_i_ and R_i_ are modeled by log-normal functions characterized by only two parameters each, the dispersion, measured in time decades and the median time, here transformed to the time to 0.1% instead of time to 50%. Table 1 gives a picture of the cancer risk over life for one person or persons born in the same year. The damage intensity over life is determined by the damage-dose numbers N_i_ and was related to measured sun tanning habits that change a bit over life [2]. In order to calculate age-standardized rates of melanoma over time, we have to set up a series of Life Matrices starting from 1860 up till present days. By parameter variation it was an easy task to determine the two functions that gave the best fit to reported data. In order to simplify the process we fixed the time to 0.1% to 100 years for the cancer risk function C, and to five years for the Repair function R so that only the two dispersion parameters had to be varied to best fit data.

**Table 1:** The Life Matrix sums vertically the partial melanoma risks (N_i_F_j_) emanating from damages (N_i_) attained each year during a person’s life and gives the total melanoma risk over life (bottom line).

						*Year*				
*Year*	*Dmg*	*Y_1_*	*Y_2_*	*Y_3_*	*Y_4_*	*Y_5_*	*Y_6_*	*Y_7_*	.	.
*Y_1_*	*N_1_*	*N_1_F_1_*	*N_1_F_2_*	*N_1_F_3_*	*N_1_F_4_*	*N_1_F_5_*	*N_1_F_6_*	*N_1_F_7_*	.	.
*Y_2_*	*N_2_*		*N_2_F_1_*	*N_2_F_2_*	*N_2_F_3_*	*N_2_F_4_*	*N_2_F_5_*	*N_2_F_6_*	.	.
*Y_3_*	*N_3_*			*N_3_F_1_*	*N_3_F_2_*	*N_3_F_3_*	*N_3_F_4_*	*N_3_F_5_*	.	.
*Y_4_*	*N_4_*				*N_4_F_1_*	*N_4_F_2_*	*N_4_F_3_*	*N_4_F_4_*	.	.
*Y_5_*	*N_5_*					*N_5_F_1_*	*N_5_F_2_*	*N_5_F_3_*	.	.
*Y_6_*	*N_6_*						*N_6_F_1_*	*N_6_F_2_*	.	.
*Y_7_*	*N_7_*							*N_7_F_1_*	.	.
.	.								.	.
.	.	*∑Y_1_*	*∑Y_2_*	*∑Y_3_*	*∑Y_4_*	*∑Y_5_*	*∑Y_6_*	*∑Y_7_*		.

To confirm the validity of the hypothesis we will use this set of Life Matricxes to calculate the world age-standardized rate over time by parameter variation and then just check if also age-specific rates over time fits the reported data without further parameter variation.

#### 2.5.2. Testing the Reduced Repair Hypothesis

Figure 2 shows the drastic change in age-specific melanoma incidence from the mid-20th century. It clearly appears that damages occurring during the first part of this century all became repaired or removed within around 30 years, since the incidence stays constant after the age of 30. The natural repair function thus should remove of damage within 30 years, while during the second part of the century, much more of the damage would stay active for longer times.

**Figure 19:**
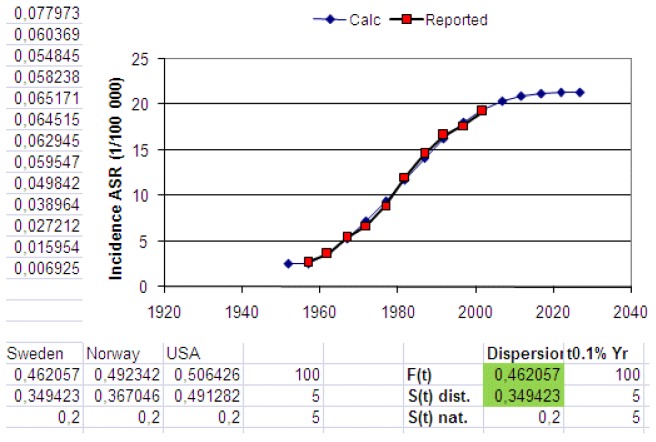
An extract from the Excel application where calculated and reported age-standardized rates have been fitted by varying the two dispersion parameters shown in green fields.

Figure 19 shows an extract from the Excel application where the calculated age-standardized rates of melanoma in Sweden by parameter variation have been fit to reported data. In the background the computer has also made all calculations of age-specific rates because they are used as the base when calculating the age-standardized rates (here for the Swedish population in 1970). A detailed description of this study on data from Sweden, Norway and the USA is given in [2].

In Figure 19 the natural dispersion is set to 0.2, while the disturbed dispersion is wider (0.35) and was found by best fit optimization as also was the case for the cancer risk dispersion (0.46) for Swedish data.

**Figure 20:**
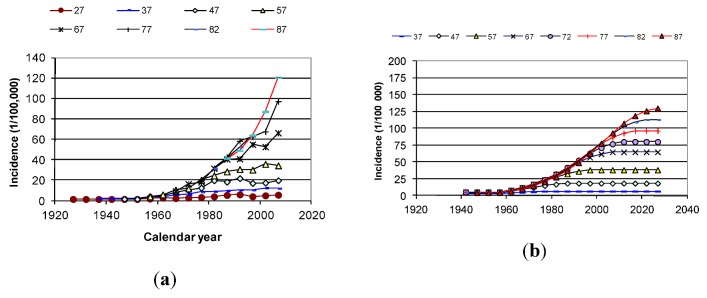
(**a**) Reported age-specific incidence, for men in Sweden [2]. (**b**) Calculated age-specific incidence for men in Sweden [2].

Figure 20a,b show reported and calculated age-specific melanoma incidence rates in Sweden based on the two parameters highlighted in Figure 19. The change from natural dispersion (0.2 decades) to disturbed dispersion (0.35 decades) was set to 1960. We have recently also looked at the mortality rates. It appears that the mortality from melanoma among men in the Nordic countries is exponentially increasing by the time lived as adult (>15 years of age) since 1955.

**Figure 21:**
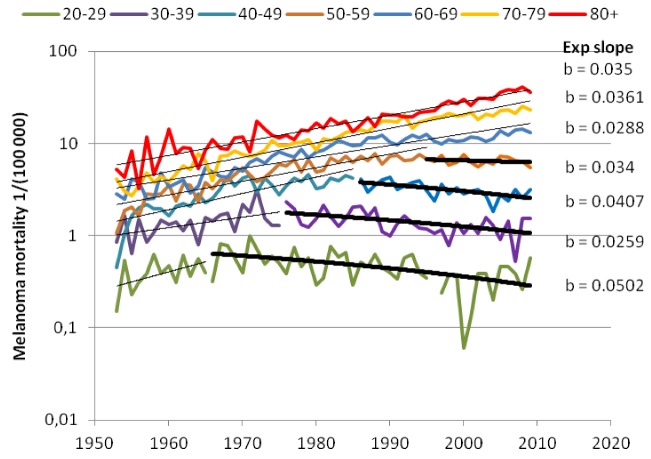
The melanoma mortality among men in the Nordic countries is increasing exponentially as a function of time lived in the Nordic environment after 1955.

Once e.g., the age group of 45 years has been living all 30 years after their 15th birthday in the post-1955 environment, the mortality instead levels off and even starts to decrease. This is clearly shown in Figure 21.

#### 2.5.3. Testing the Sun-Tanning Hypothesis

According to the Swedish Radiation Safety Authority it is assumed that the habit of sun-tanning started to increase from 1930 up till 1980, when the warnings regarding the danger of the sunshine escalated. In order to model this scenario we assumed a linearly increasing exposure to sun originated cell damages during those 50 years. The repair rate was assumed to be intact, *i.e*., the dispersion was kept constant at 0.2 decades.

**Figure 22:**
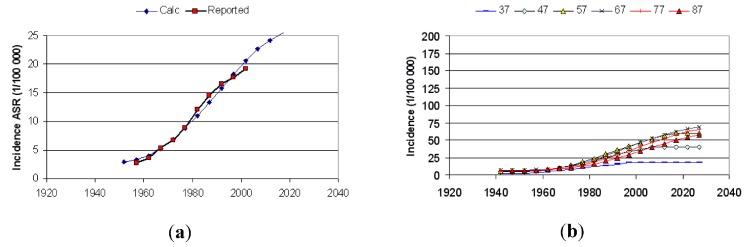
(**a**) Calculated and reported ASR assuming increasing skin damage rates between 1930–1980 [2]. (**b**) Calculated age-specific rates based on increasing sun-tanning habits [2].

Figure 22a shows the best fit of calculated to reported age-standardized rates, which is not entirely convincing. The best fit was obtained for the case when sun tanning in 1980 was around 10 times as intense as in 1930. Figure 22b finally shows the corresponding calculated age-specific rates that do not mathematically match reported data [2] (Figure 20a) at all. Was there a sudden increase in sun tanning habits by a factor of 10 already in 1930? Also this hypothesis could easily be tested, but still has to be done.

## 3. Discussion

This review of facts regarding melanoma strongly indicates that the main problem is not too many skin cell damages, but rather too few repairs, where repair here refers to both DNA repair, apoptosis and/or influence from immunosurveillance. We know that healthy smokers have better DNA repair capacity (DRC) than healthy non-smokers [15], but also that smokers who have got lung cancer have a reduced DRC compared with non-smokers. Outdoor workers tend to have a reduced risk of getting melanoma compared with indoor workers. While a reduced DRC has been shown to be a risk factor in several cancers, the studies by Landi *et al*. [16] and Matta *et al.* [17] did not show a statistical significant difference between melanoma cases and controls. This could be logical if the repair work is disturbed by skin currents only during night time, thus the DRC *per se* works fine if it is tested undisturbed. It would be quite difficult to suddenly increase the melanoma incidence or mortality simply by sitting in the sunshine for much longer times than before, and if the increase was due to travel to sunnier resorts from the beginning of the 60s, then the melanoma incidence would have had to be over 100 times higher among those who could afford the tickets than among the rest of the population, but no such strange difference has been reported. Exposure to UVA and UVB radiation has been found to be a major contributor to the initiation of melanoma [18], but has not been shown to explain the trend changes since the mid-20th century. Part of the increasing incidence rates might be explained by increased awareness and screening, and possibly over-diagnosis, but this does not explain the total increase [19].

**Figure 23:**
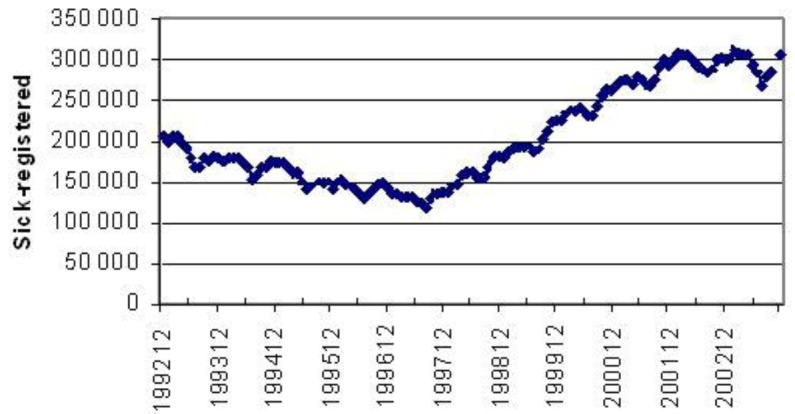
Every summer the number of sick registered in Sweden drops. In 1997 the general trend got worsened.

Actually, sun tanning supports the production of an important hormone, vitamin D, and the net effect of the sunshine is always that people are healthier in the summer time than in the winter. The increase at winter time in both sick-days and mortality is also due to respiratory tract infections that always are more common in that time of the year. Figure 23 gives the number of people on sick leave in Sweden per month.

It seems peculiar to the authors that the alternative explanation of reduced efficiency of the repair mechanisms never has been addressed in the scientific literature, despite the fact that data have been available for many years right in front of our eyes. When the problem of increasing melanoma rates became obvious, the authorities pointed at the sun, and suddenly a multi-billion $ market opened for the sun-block cream industry. At the same time the telecom market flourished and the air became filled with many other types of radiation apart, from UV-radiation from the Sun. The economic interests in blaming the Sun for the increasing rates of melanoma became astronomical, and lobbying experts guided our politicians and authorities along the ways that best suited their interests.

**Figure 24:**
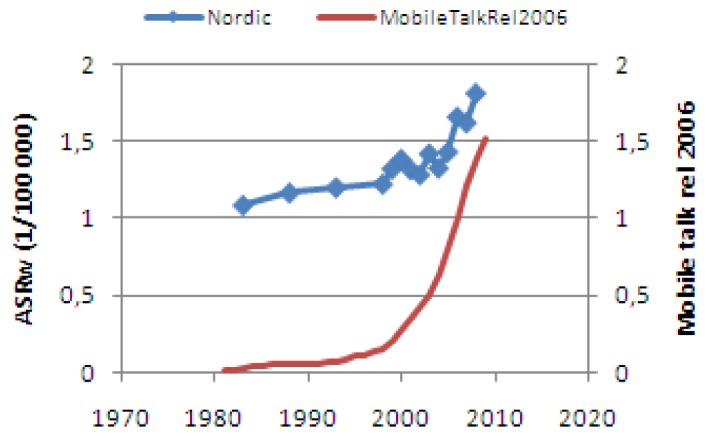
Since 2005 the incidence of melanoma in the head/face region is increasing in all Nordic countries. Since 2000 the use of mobile phones has increased by almost ten times [21].

From Figure 9 we can see that melanoma has been on the rise again since 2005. A more detailed investigation shows that a reasonable part of this increase happens to take place in the face or on the head area. At the same time the use of mobile phones has merely exploded and it is an obvious task to investigate if there is an association or even a causative effect. A study of the face/head melanoma rates in the Nordic countries shows that all countries suffer from the same negative trends; head melanoma is increasing! (see Figure 24). Incidence and mortality data can easily be downloaded from the NORDCAN database [20].

The fact that also the incidence of breast cancer and lung cancer can be associated to melanoma strongly supports the hypothesis of a common factor. Both breast cancer and melanoma have a left dominance of around 10%, indicating that a good and simple way of reducing the cancer risk is to get rid of the metal spring mattress and only sleep on non-reflecting futons or soft mattresses on wooden slats [11]. Lung cancer has a right dominance of 25% [14], but since the left lung volume is 25% less than the right lung, we have still a 6% relative overweighting for the left lung.

There have been efforts to correlate melanoma risk to temperature in a country, since many countries show higher incidence in their more southern parts. However, these countries most often are more densely populated in these areas and also better covered by main broadcasting transmitters. An exception is e.g., France, where the melanoma incidence is higher up north than in the south, quite according to population and transmitter density.

## 4. Conclusions

Based on the findings, facts and tests of hypotheses presented in this review, the main conclusion is that the melanoma epidemic is a result of the modern man-made environment that forces us to live and sleep in invisible but still unhealthy electromagnetic smog.

There are many ways to improve the situation, both by measures taken in the home and by the society, but we need to stand up to the strong economic interests that rather want us to continue buying expensive spring beds, new mobile phones, and applying layers of sun cream over the whole body. The medical industry has no interest in reduced needs for cancer treatment and medications, and the various cancer foundations will support research on cancer treatment and medicines, but are less likely to support cancer prevention research.

There are several simple and quite inexpensive studies that should be performed to further substantiate the relevance of our hypothesis. One is to make an enquiry among still smoking and still healthy elderly (80+) about their use of metal spring mattresses to compare that with standard beds among lung cancer patients. Another test would be to compare bed standards between breast cancer patients and healthy controls, and the same for melanoma.

The very large difference in melanoma risk between Japan and Sweden can certainly not be explained by a pigmentation difference since the cancer risks of Japanese people increase once they move to Western countries, and furthermore, why do their children grow so fast once they have moved to e.g., the USA? Body length and cancer is certainly a research area worthy of more attention than paid today.
